# Key factors dominating the neural coding preference to 1/f signal

**DOI:** 10.1186/1471-2202-16-S1-P296

**Published:** 2015-12-18

**Authors:** Boqiang Fan, Wen Zhang, Shanglin Zhou, Yuguo Yu

**Affiliations:** 1 School of Life Sciences, the State Key Laboratory of Medical Neurobiology and Institutes of Brain Science, Fudan University, Shanghai, 200433, Republic of China

## 

**Figure 1:**
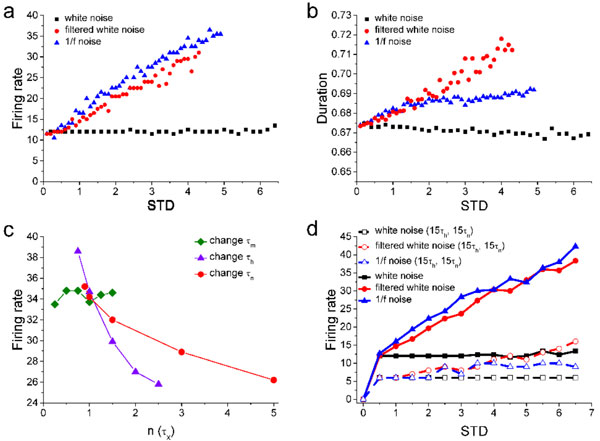
**Interactions among neuron kinetics and signal statistics**. a) Firing rate vs signal STD for white noise, filtered white noise and 1/f noise. b) Half-height duration of action potentials vs signal STD for white noise, filtered white noise and 1/f noise. c) For a fixed input 1/f signal (STD = 1), firing rate change as a function of sodium activation time constant (τm), inactivation time constant (τh) and potassium activation time constant (τn). d) firing rate vs signal STD for , In the situation with both τh and τn being increased to 15 times larger.

Experiments have demonstrated that cortical and sensory neurons prefer to response to signals with characteristics of long-term correlation or 1/f noise feature better than signals with no correlation like white-noise-type [1]. In order to study the underlying mechanism, we built up a cortical neuronal model [2] based on Hodgkin-Huxley theory to study the correlations between neuron kinetics and signal statistics. Interestingly, we observed that (see Figure 1a,b) white-noise-type signal (cutoff frequency >10000Hz) is hard to induce action potentials unless signals with very strong intensity while 1/f signal and low-pass filtered white noise type signal (cutoff frequency <1000Hz) can easily induce action potentials with high firing rate at low signal intensity (quantified by signal standard deviation STD), see Figure 1a. Moreover, the half-height duration of action potentials is also varying with more sensitivity to 1/f signal or filtered white noise than pure white noise (Figure 1b). In addition, neural firing rate and spike duration are more sensitive to 1/f signal than filtered white noise. This is a clear positive evidence of neuronal coding preference to signals with long-term correlations.

To further reveal the key factors dominating the preference of neuronal dynamics to colored and white noise, we systematically varied the values of Na+ and K+ channel time constants and channel rate constants. Figure 1c showed that the firing rate doesn't change much as a function of sodium activation time constant (τm) for a given 1/f signal (STD = 1). However, the firing rate decrease dramatically with an increase of sodium inactivation time constant (τh) or potassium activation time constant (τn) for this signal. For very large τh and τn, neuronal response dynamics start to be saturate for all signals, see Figure 1d. These results indicate that both sodium inactivation time constant (τh) and potassium activation time constant (τn) may be the key factors dominating neural coding preference to signals with different correlated statistic features.

In sum, this study demonstrated that ionic channel time constant of sodium inactivation and potassium activation may be the dominating factors accounting for the neuronal spiking sensitivity favorable to signals with temporal correlations.
